# Preparation of Self-Assembled, Curcumin-Loaded Nano-Micelles Using Quarternized Chitosan–Vanillin Imine (QCS-Vani Imine) Conjugate and Evaluation of Synergistic Anticancer Effect with Cisplatin

**DOI:** 10.3390/jfb14100525

**Published:** 2023-10-18

**Authors:** Sasikarn Sripetthong, Sirinporn Nalinbenjapun, Abdul Basit, Suvimol Surassmo, Warayuth Sajomsang, Chitchamai Ovatlarnporn

**Affiliations:** 1 Department of Pharmaceutical Chemistry, Faculty of Pharmaceutical Sciences, Prince of Songkla University, Hat Yai 90112, Thailand; sasikarn.spt@gmail.com (S.S.); nasirin109@hotmail.com (S.N.); abdulbasit.pharmd@gmail.com (A.B.); 2 Drug Delivery System Excellent Center, Faculty of Pharmaceutical Sciences, Prince of Songkla University, Hat Yai 90112, Thailand; 3 Nano-Delivery System Laboratory, National Nanotechnology Center, National Science and Technology Development Agency, Thailand Science Park, Pathum Thani 12120, Thailand; suvimol@nanotec.or.th (S.S.); warayuth@nanotec.or.th (W.S.)

**Keywords:** nano-micelles, quarternized chitosan–vanillin conjugate, curcumin, cisplatin, lung cancer, cytotoxicity evaluation

## Abstract

Nano-micelles are self-assembling colloidal dispersions applied to enhance the anticancer efficacy of chemotherapeutic agents. In this study, the conjugate of quarternized chitosan and vanillin imine (QCS-Vani imine) was synthesized using the reaction of a Schiff base characterized by proton-NMR (^1^HNMR), UV-Vis spectroscopy, and FT-IR. The critical micelle concentration (CMC), particle size, and zeta potential of the resulting product were determined. The QCS-Vani imine conjugate was used as a carrier for the development of curcumin-loaded nano-micelles, and their entrapment efficiency (%EE), drug-loading capacity (%LC) and in vitro release were investigated using HPLC analysis. Moreover, the nano-micelles containing curcumin were combined with various concentrations of cisplatin and evaluated for a possible anticancer synergistic effect. The anticancer activity was evaluated against lung cancer A549 and mouse fibroblast L929 cell lines. The percent yield (%) of the QCS-Vani imine conjugate was 93.18%. The curcumin-loaded QCS-Vani imine nano-micelles were characterized and found to have a spherical shape (by TEM) with size < 200 nm (by DLS) with high %EE up to 67.61% and %LC up to 6.15 ± 0.41%. The loaded lyophilized powder of the nano-micelles was more stable at 4 °C than at room temperature during 120 days of storage. pH-sensitive release properties were observed to have a higher curcumin release at pH 5.5 (cancer environment) than at pH 7.4 (systemic environment). Curcumin-loaded QCS-Vani imine nano-micelles showed higher cytotoxicity and selectivity toward lung cancer A549 cell lines and exhibited lower toxicity toward the normal cell (H9C2) than pure curcumin. Moreover, the curcumin-loaded QCS-Vani imine nano-micelles exhibited an enhanced property of inducing cell cycle arrest during the S-phase against A549 cells and showed prominently induced apoptosis in lung cancer cells compared to that with curcumin. The co-treatment of cisplatin with curcumin-loaded QCS-Vani imine nano-micelles presented an enhanced anticancer effect, showing 8.66 ± 0.88 μM as the IC_50_ value, in comparison to the treatment with cisplatin alone (14.22 ± 1.01 μM). These findings suggest that the developed QCS-Vani imine nano-micelle is a potential drug delivery system and could be a promising approach for treating lung cancer in combination with cisplatin.

## 1. Introduction

Lung cancer is a serious public health problem and a leading cause of cancer death worldwide [1]. It is the most commonly diagnosed form of malignancy globally, accounting for 11.4% of all cancer diagnoses. Moreover, it is the leading cause of mortalities due to cancer, accounting for 18% of all cancer mortalities [2]. It is reported that about 75% of lung cancer cases at the advanced stage result in high patient morbidity due to the ineffectiveness of the existing treatments. A significant increase in the occurrence of lung cancer was noted in 2022; 12.3% of cases of all cancers were reported to be lung cancer in the United States, with an estimated fatality rate of 21.4% for newly reported lung cancers. According to the literature [3,4,5], individuals suffering from lung cancer normally have a 5-year survival rate on average. Early-stage patients with lung cancer generally present with no apparent symptoms, whereas middle- or late-stage lung cancers are diagnosed after clinical presentations such as chest pain, hemoptysis, cough, and dyspnea [6]. Conventional chemotherapy is a widely used approach for cancer treatment; however, it often exhibits limited efficacy and significant side effects. To overcome these limitations, researchers have focused on creating adjunctive chemotherapy approaches to increase currently available treatment protocols, which may decrease the side effects and toxicity level without compromising the therapeutic efficacy. One promising approach is combination therapy, where two or more drugs with distinct mechanisms of action are used simultaneously to achieve a synergistic effect. This approach aims to enhance the overall therapeutic outcome by targeting multiple pathways involved in cancer development and progression. In this context, the combination of cisplatin, a widely used chemotherapeutic agent, with curcumin, a natural compound with potent anticancer properties, could be a valuable approach for overcoming the prevalence of lung cancers.

Cisplatin, a platinum-based drug, exerts its cytotoxic effect by forming DNA crosslinks and inhibiting cancer cell proliferation [7]. However, its clinical utility is limited due to the development of resistance and severe side effects. On the other hand, curcumin (diferuloylmethane, Figure S1) is the most crucial active compound derived from the turmeric rhizome *Curcuma longa* [8,9,10]. Curcumin causes growth arrest and apoptosis in lung cancer cell lines and inhibits lung cancer cell invasion and metastasis through the tumor suppressor HLJ1 [11,12]. Curcumin has been one of the most commonly used Ayurvedic medicines for a long time due to its multiple properties, such as anti-inflammatory, antioxidant, antiseptic, and analgesic, as well as the safety and non-toxic nature of the compound [13,14]. Currently, a lot of reports have been published on the anticancer potential of curcumin with elucidation of its probable anticancer mechanisms, such as apoptosis and cell cycle arrest, as well as its inhibitory effect on tumor proliferation and metastasis. The pharmacological effects of curcumin in lung cancer have been found by the modulation of several miRNAs, such as the downregulation of oncogenic miR-21 and upregulation of onco-suppressive miR-192-5p and miR-215 [15]. Curcumin has anti-metastatic potential by decreasing the invasiveness of cancer cells involved in the p-ERK and MEKK3 signaling pathways, leading to the inhibition of MMP-2 and MMP-9 and resulting in the inhibition of MMP-2 and -9 in human lung cancer A549 cells [16]. The versatile properties of curcumin include the regulation of oncogenesis factors, like RGR-1, p53, bcl-XL, and c-myc; transcription factors, such as NF-kB, AP-1, and STAT-3; protein kinases, such as MAPK; and various enzymes, such as cyclo-oxygenase and lipoxygenases. These properties make curcumin a suitable candidate for its employment in the adjunct treatment of solid tumors [17,18]. However, certain limitations, such as poor solubility, poor oral absorption, and rapid metabolism, have limited its clinical applications [19]. To cope with these issues, various investigators have unveiled the successful application of drug delivery systems that can enhance the stability, solubility, and targeted delivery of anticancer agents. One such system is the use of a vanillin–chitosan conjugate as a carrier, which received the attention of researchers as a promising carrier for the delivery of various therapeutically active agents, as such systems have multiple advantages, such as muco-adhesiveness, biodegradability, and compatibility with biological systems. Vanillin, a promising natural compound derived from vanilla beans, acts as a hydrophobic moiety on the chitosan backbone for nano-micelle formation. Chitosan is a polysaccharide derived from crustacean shells and has been extensively used in target drug delivery systems [20,21,22]. The most common nano-carriers from chitosan for encapsulating therapeutic drugs including liposomes, polymeric nanoparticles/nano-micelles, and inorganic nanoparticles for anticancer activity enhancement were successfully developed [23,24,25].

The main purpose of the current research was to prepare a quarternized chitosan oligosaccharide–vanillin imine (QCS-Vani imine) polymer conjugate and use it for the formulation of curcumin-loaded nano-micelles. The nano-micelles were selected as drug delivery systems due to their unique properties such as structural stability (both thermodynamic and kinetic), ability to solubilize hydrophobic drugs, pH sensitivity, muco-adhesiveness, specific binding ability, and most importantly, the advanced physicochemical properties due to the presence of amphiphilic monomers in their structures [26]. The obtained nano-micelles were investigated for their physicochemical properties, loading capacity, pH sensitivity, drug release, and cytotoxicity activity against lung cancer and normal cell lines. The combination of cisplatin with curcumin-loaded QCS-Vani imine nanoparticles holds significant promise as a synergistic anticancer approach. This system can be applied as supplemental therapy with other anticancer agents which will induce its anticancer effect and provide synergism in combination with anticancer agents. The use of quarternized chitosan can increase the specificity of the vehicle toward the tumor site. By exploiting the unique properties of each component, this combination therapy offers the potential for enhanced efficacy, reduced toxicity, and improved patient outcomes. The present study aims to shed light on the underlying mechanisms and therapeutic potential of this novel approach, ultimately paving the way for its clinical translation and application in cancer patients.

## 2. Materials and Methods

### 2.1. Chemicals

Materials were acquired from the following sources: chitosan (80 kDa with 85% DD) from Seafresh Chitosan (Lab) Co., Ltd., Chumporn, Pak Nam, Thailand; analytical grade benzaldehyde from LOBA CHEMIE PVT.Ltd., Mumbai, Maharashtra, India; analytical grade hydrochloric acid (37%), sodium hydroxide, glacial acetic acid, ethanol, methanol, isopropanol, and dimethyl sulfoxide (DMSO) from Labscan Asia, Bangkok, Thailand; and glycidyl trimethyl ammonium chloride (GTMAC) and cisplatin from Sigma-Aldrich, Burlington, MA, USA. Food-grade vanillin was acquired from Hong Huat Company Limited, Bangkok, Thailand. Human lung carcinoma cell line (A549), rat embryonic cardiomyocyte (H9C2) and mouse fibroblast cell line (L929) were purchased from ATCC^®^, Manassas, VI, USA. High-glucose and low-glucose DMEM (Dulbecco’s modified eagle medium), trypsin-EDTA (25%) and FBS (fetal bovine serum) were purchased from Gibco^®^ by Life Technologies, Toronto, ON, Canada. The MTT (3-[4,5-dimethylthiazol-2-yl]-2,5-diphenyltetrazo-dium bromide) reagent was acquired from Invitrogen TM Molecular Probes^®^, Eugene, OR, USA and PBS (phosphate-buffered saline of 7.4 pH) was purchased from Sigma-Aldrich, Darmstadt, Germany.

### 2.2. Preparation of QCS-Vani Imine Conjugate

#### 2.2.1. Preparation of Glycidyl Trimethyl Ammonium–Quarternized Chitosan (GTMAC-QCS)

Benzyl-chitosan (B-CS) was synthesized using the previously reported method of Li et al. [27] with a few modifications. Briefly, in this method, 700 mL of 2% acetic acid was used to make a solution of chitosan (CS, 80 kDa) (10 g, 0.059 mol eq/GlcN) at 70 °C using a magnetic stirrer. After 24 h, 60 mL of benzaldehyde solution (62.61 g (0.59 mol) in 100 mL methanol) was added dropwise into the CS solution. The reaction was refluxed overnight at 70 °C with continuous stirring and then cooled down to room temperature. The pH of the mixture was adjusted to 7.0 by the addition of a 3 M sodium hydroxide (NaOH) solution. After this, the mixture was filtered and washed with methanol to remove the unreacted benzaldehyde. The product was dried at 60 °C for 24 h.

Benzyl-glycidyl trimethylammonium-chitosan (B-GTMAC-QCS) was prepared by the previously reported method of Sasaki, Sunagawa, Takahashi, Imaizumi, Fukuda, Hashimoto, Wada, Katanasaka, Kakeya and Fujita [20] with slight modifications as follows: B-CS (11.59 g, 45.00 mmol) was suspended in isopropanol (150 mL). Then, glycidyltrimethylammonium chloride (GTMAC) (36.23 mL, 40.95 g, 270.00 mmol, 1:6) was poured into the mixture and subjected to gentle stirring for 24 h at 85 °C. After this, the obtained product was filtered and washed with EtOH (AR) and then vacuum dried at 60 °C.

The deprotection process was performed by dispersing B-GTMAC-QCS (28 g) in 0.25 M HCl and ethanol (300 mL) with continuous gentle stirring for a time period of 24 h. Then, the mixture was neutralized using 1% Na_2_CO_3,_ which resulted in a product that was precipitated using acetone and filtered, which was followed by the addition of 400 mL of water to make it dissolve. This solution was then subjected to the process of dialysis for three days against distilled water for the purpose of removing impurities. Then, the final product (GTMAC-QCS) was obtained after carrying out lyophilization.

#### 2.2.2. Vanillin Conjugation on GTMAC-QCS by Schiff Base Reaction

QCS-Vani imine conjugate was synthesized using the previously reported method of Ma, Wang, He and Tang [22]. The conjugate was synthesized through the Schiff base reaction. The GTMAC-QCS (1.5 g, 8.85 meq/GlcN) was dissolved in water (150 mL) at 70 °C using a magnetic stirrer. Subsequently, 0.67 g (4.425 mmol) vanillin was dissolved in ethanol (50 mL) and added dropwise into the GTMAC-QCS solution. The reaction was refluxed overnight at 70 °C with constant stirring and cooled down to room temperature. This solution was then subjected to the process of dialysis for three days against distilled water to remove the impurities. Then, the final product (QCS-Vani imine) was obtained after carrying out lyophilization

### 2.3. Characterization of QCS-Vani Imine Conjugate

#### 2.3.1. ^1^H NMR, FTIR and Spectrophotometric Analysis

The NMR analysis of the significant intermediates and final conjugated product was carried out using the instrument NMR, ADVANCE NEO 500 MHz, Bruker, Germany, using previously established protocols with little modifications [28]. The sample was prepared in D_2_O. The sample of chitosan for the analysis was prepared in deuterated methanol spiked with D_2_O and acetic-D_3_.

Functional group analysis of the conjugate was characterized by using an FT-IR spectrometer (Spectrum One, Perkin Elmer Ltd., Buckinghamshire, Beaconsfield, UK). All the samples were put in a desiccator and dried overnight. Then, about 10% of each sample was mixed with IR-grade potassium bromide (KBr) to make pellets. The FT-IR spectrum of every sample was acquired in at least 16 scans using a range from 400 to 4000 cm^−1^.

A UV-visible spectrophotometer (Cary 60, Agilent) was also used to confirm the structure of vanillin that was attached to the backbone of the quarternized chitosan. The samples analyzed were prepared as follows: 0.4 mg/mL of QCS-Vani imine in distilled water, chitosan in 2% acetic acid and 2 µg/mL of vanillin in ethanol. The range of 200–500 nm was used for recording the absorbance spectrum of every sample.

#### 2.3.2. Degree of Substitution of Quaternary Groups (DQ) on Glycidyltrimethylammonium–Chitosan (GTMAC-QCS)

The degree of quaternary substitution on GTMAC-QCS was determined by the potentiometric titration [29]. In brief, GTMAC-QCS (30 mg) was dissolved in distilled water (60 mL); then, the resulting solution was titrated with a standard solution of 0.01 M AgNO_3_ until it reached the endpoint. The volume of 0.01 M AgNO_3_ was recorded (V_AgNO3_) and used in the calculation of the degree of quaternization (*DQ*) using the below-given equation.

$$DQ=\frac{A}{A+\frac{\left(w-\left(A\;\times \;M2\right)\right)}{M1}}$$

where

$A=V_{AgNO_{3}}\;\times \;C_{AgNO_{3}}\;\times \;10^{-3}$;
$V_{AgNO_{3}}$ = volume of 0.01 M AgNO_3_ at end point (mL);
$C_{AgNO_{3}}$ = molarity of AgNO_3_ (M);
w = mass of Q-CS (g), *M*_1_ = molecular weight of glucosamine = 169.4; and
*M*_2_ = molecular weight of QCS = 152.6.

#### 2.3.3. Degree of Substitution of Vanillin on QCS-Vani Imine Conjugate

The degree of substitution of vanillin on the chitosan network in QCS-Vani imine was determined by the acid hydrolysis method [22] as described. QCS-Vani imine (15 mg) was placed in a 50 mL volumetric flask and treated in 1 M HCl (40 mL) for 24 h at 37 °C using a shaking incubator. Then, the solution was adjusted to 50 mL, and the resulting solution was determined for the released vanillin by a UV-vis spectrophotometer at 280 nm. The degree of substitution (*DS*) was determined using the below equation.

$$DS=\left[\frac{\left(\frac{mV}{Mv}\right)}{\frac{\left(mCV-mV\right)}{Mcv}}\right]\times 100$$

where *mV* = mass of vanillin, *mCV* = mass of QCS-Vani imine conjugate, *Mv* = molecular weight of vanillin and *Mcv* = molecular weight of VA-CS (per unit).

### 2.4. Preparation of Nano-Micelles

#### 2.4.1. Preparation of Curcumin-Loaded QCS-Vani Imine Nano-Micelles

The preparation of curcumin-loaded QCS-Vani imine nano-micelles involved dissolving QCS-Vani imine (30 mg) in DMSO (10 mL, 90%) at room temperature. Then, various concentrations (0.1, 0.3, 0.4 and 0.45 mg/mL) of curcumin solutions were added to the QCS-Vani imine solution (Table 1). The final solution of each sample was adjusted to 10 mL with DMSO. The solution was sonicated in an ice bath for 5 min using a probe sonicator (40 amplitude, the pulse function was pulse on 50.0 s and pulse off 10.0 s). Finally, the solution was subjected to dialysis for 18 h using distilled water and a dialysis bag with MW. The cut-off was equal to 3500 [30,31].

#### 2.4.2. Preparation of Curcumin-Loaded QCS-Vani Imine Nano-Micelles Powders

The powder form of curcumin-loaded QCS-Vani imine nano-micelles was obtained by the freeze-drying method [32]. QCS-Vani imine solution (4 mg/mL) was prepared in 90% DMSO. Curcumin solutions were prepared separately in DMSO at a concentration of 10 mg/mL, which was followed by the slow and steady addition of the solutions of curcumin to the solution of QCS-Vani imine at 25 °C with constant stirring, which resulted in a clear solution. The solutions were then sonicated in an ice bath for 5 min using a probe sonicator (40 amplitude, the pulse function was pulse on 50 s and pulse off 10.0 s). The resulting solution was poured into the dialysis bag, and dialysis was performed using distilled water at 25 °C for 18 h. The micelles solution was then added by glycine to have a final concentration of 1% w/v before carrying out the freeze drying to obtain nano-micelle powders.

### 2.5. Characterization of Nano-Micelles

#### 2.5.1. Critical Micelle Concentration (CMC)

The CMC determination for QCS-Vani imine nano-micelles was conducted using the technique of fluorescence spectroscopy [33]. Initially, solutions of QCS-Vani imine at various concentrations ranging from 0.002 to 1 m/mL were prepared in test tubes using 4 mL of distilled water followed by the addition of pyrene (0.1 mM) in acetone (10 µL) to each test tube. This was then subjected to sonication for 2 h at 25 °C for 15 min followed by heating at 50 °C for 2 h and finally stored overnight to equilibrate in a light-deprived room. A microplate reader (Thermo-Scientific) was used to determine the fluorescence of these samples at 335–500 nm. The shift in vanillin hydrophobic microdomains was observed and monitored by changes in the ratio of the intensity (I_1_/I_3_) at a wavelength of 390 nm (I_3_) and 375 nm (I_1_) through the emission spectra. Subsequently, CMC was computed by fitting the plot of I_1_/I_3_ (intensity ratio) against the logarithm of QCS-Vani imine concentrations. The concentration of QCS-Vani imine was expressed in terms of mg/mL.

#### 2.5.2. Size and Zeta Potential

The QCS-Vani imine nano-micelles were subjected to the determination of size, zeta-potential and size distribution through a Zetasizer Nano ZS (Zetasizer Nano ZS, Malvern, UK) [34].

#### 2.5.3. The Entrapment Efficiency (%EE) and Loading Capacity (%LC) Determination of Curcumin-Loaded QCS-Vani Imine Nano-Micelles

The percent entrapment efficiency (%EE) and percent loading capacity (%LC) of curcumin-loaded QCS-Vani imine nano-micelles were determined by using the HPLC method as described [34]. The %EE of curcumin in curcumin-loaded QCS-Vani imine nano-micelles was analyzed by mixing the nano-micelle solution with dimethyl sulfoxide (DMSO) to give a clear solution. The powdered nano-micelles were accurately weighed and mixed in DMSO and H_2_O (9:1 *v*/*v*) to give a clear solution for %LC determination. All samples were filtered through a 0.22 µm syringe filter before the HPLC analysis. The below equation was used for the calculation of %*EE*.

$$\%EE=\frac{amount\;of\;Cur\;encapsulated\;in\;micelles}{wt\;of\;Cur\;added\;in\;micelles\;preparation}\times 100$$

%*LC* of curcumin was calculated through the below-given equation.

$$\%LC\;\frac{\;Amount\;of\;Cur\;encapsulated\;in\;micelles}{Wt\;of\;curcumin-loaded\;QCS-Vani\;imine\;micelles}\times 100$$

All the performances were carried out in triplicate.

#### 2.5.4. Morphology Observation

The morphological observations of the QCS-Vani imine nano-micelles were carried out using transmission electron microscopy (TEM) in accordance with the previously established protocols [34]. The samples were stained with a 2% uranyl acetate solution (*w*/*v*) and then placed on copper grids (200 mesh) for viewing.

### 2.6. Differentials Scanning Calorimetry (DSC) Analysis

The thermal characteristics of the samples were analyzed with a DSC (Differential Scanning Calorimeter (Perkin Elmer DSC 8000, Waltham, MA, USA)). First, 5 mg of the sample was kept in a properly sealed aluminum pan. Then, the samples were subjected to heating at a rate of 10 °C/min in the range of 50–350 °C under nitrogen purge at 20 mL/min.

### 2.7. Stability Study of the Curcumin-Loaded QCS-Vani Imine Nano-Micelle Powders

The obtained nano-micelles were tested for their stability during storage. The nano-micelles were kept in a well-closed light-preventive container under two different temperatures (4 °C and 30 °C) for 16 weeks. At each sampling time point, the stored nano-micelles sample (10 mg) was taken and dispersed in 25 mL of distilled water to obtain a nano-micelles solution. The resulting solutions were then subjected to evaluation for their sizes and zeta potential values using a Zetasizer Nano ZS, Malvern, UK, and the remaining encapsulated curcumin using HPLC. All tests were performed in triplicate.

### 2.8. Study of In Vitro Drug Release

The release profile of curcumin from QCS-Vani imine nano-micelles was evaluated using the method of dialysis against the solutions of phosphate buffer of pH 5.5 (simulated tumor environment) and pH 7.4 (blood) at 37 °C. Curcumin (25 mg) and 5 mL of QCS-Vani imine nano-micelles (containing 900 µg of curcumin) were put separately in a dialysis bag made up of cellulose (molecular cut-off 3500). Each dialysis bag was then placed in a beaker that was already containing 50 mL of the test buffer medium (containing 0.5% Tween-80). The beakers were then kept in an orbital shaker (110 rpm, 37 °C). Samples (1 mL) were collected at specified time intervals (h), i.e., 1, 2, 3, 4, 6, 9, 12, 24, 32, 48, 72, 96, 120, 144, and 168 h, which were followed by replacement with the same volume of fresh media (37 °C). The content of the curcumin release was analyzed by HPLC [35]. The analysis was performed in triplicate.

#### High-Pressure Liquid Chromatography (HPLC) System

The HPLC system used consisted of a Chromaster 5110 pump and a Chromaster 5430 Diode Array Detector (DAD). The Chromstar 60 MPa system was used to monitor and process the output signal. The column specifications were as follows: 5 µm ODS (JONE chromatography, 250 mm × 4.6 mm). The mobile phase used was composed of acetic acid (2%) and acetonitrile (analytical grade) combined in a ratio of 40:60 (*v*/*v*). The flow rate was adjusted to 1.5 mL/min, and the injection volume was 10 µL. The wavelength used for detection was 428 nm.

### 2.9. Evaluation of Cytotoxic Potential

#### 2.9.1. Cell Culture

The human lung carcinoma cell line (A549) and rat embryonic cardiomyocyte (H9C2) were procured from the Faculty of Pharmaceutical Sciences, Prince of Songkla University (PSU), Hat Yai, Songkhla, Thailand. The cell lines were maintained at 37 °C, 5% CO_2_ and a relative humidity of 90%. Dulbecco’s Modified Eagle Medium (DMEM) with low glucose and DMEM with high glucose with 10% FBS (fetal bovine serum), respectively, were used for the maintenance of the cell lines.

#### 2.9.2. 3-[4,5-Dimethylthiazol-2-yl]-2,5-Diphenyl Tetrazolium Bromide (MTT) Assay

The cytotoxic potential of various samples was evaluated using an in vitro 3-[4,5-dimethylthiazol-2-yl]-2,5-diphenyl tetrazolium bromide (MTT)-based colorimetric assay. Once the cells reached 80% confluence, the subculturing of monolayers was initiated. The process was carried out with trypsinization, which was followed by seeding the cells at a density of 2 × 10^4^ cells per well with the addition of 100 µL of suspended cells in complete DMEM. The exponentially growing cells were washed out after a period of 24 h. The washing step was repeated two times with PBS of pH 7.4, which was followed by incubation with freshly prepared medium only (control), medium containing curcumin, blank QCS-Vani imine nano-micelles, and curcumin-loaded QCS-Vani imine nano-micelles for 24 h. The solution of MTT was prepared by adding 100 µL of 0.5 mg/mL MTT reagent in PBS and added to evaluate the survival of the cells. The incubation was carried on for a further 4 h with the addition of 100 µL of DMSO. After this, the optical absorbance of the MTT solution above the cell layers was estimated using a wavelength of 550 nm. Using the equation below, the percent cell viability was computed. The results of the cytotoxicity potential were represented in terms of IC_50_ values.

$$\mathrm{Cytotoxicity}\;\left(\%\right)=\frac{\left(Ab_{550\;control}-Ab_{550\;sample}\right)}{Ab_{550\;contol}}\times 100$$

### 2.10. Cellular Uptake Using Confocal Laser Scanning Microscopy (CLSM)

Cellular uptake of pure curcumin and curcumin-loaded QCS-Vani imine nano-micelles was evaluated by using confocal laser scanning microscopy (CLSM). The lung cancer cell lines (A549) were seeded (2 × 10^5^ cells per well) onto glass coverslips, which were properly sterilized and incubated in a 6-well plate. After this, the 5 µM curcumin and curcumin-loaded QCS-Vani imine nano-micelles containing 5 µM curcumin were added and incubated for 2, 6 and 24 h under 5% carbon dioxide at 37 °C. Following incubation, the QCS-Vani imine nano-micelles were washed with a phosphate buffer of pH 7.4, the washing was repeated two more times, and then the cells were fixed with cold ethanol (70%) for 10 min. For visualization of the cellular nuclei, Hoechst 33342 dye (2’-[4-ethoxyphenyl]-5-[4-methyl-1-piperazinyl]-2,5’-bi-*1H*-benzimidazole trihydrochloride trihydrate) was added. Hoechst 33342 was diluted using sterilized water to the level of 1:1000 *v*/*v* and added to the fixed cell in every well at a concentration of 2 mL, which was followed by incubation at 37 °C for 30 min in a light-deprived chamber. This was followed by repeated washing, which was carried out using a phosphate buffer of pH 7.4. For visualization of the cells CLSM (Zeiss LSM 800, Zeiss, Germany), Programme ZEN 2.3 (Zeiss Microscopy GmbH, Germany) was employed with the utilization of the 63×objective lens and Airy scan detector.

### 2.11. Cell Apoptosis Study Using Flow Cytometry

The cell apoptosis study was conducted through the application of the Annexin V-FITC/propidium iodide (PI) double-staining approach to identify both necrotic and apoptotic populations. The lung cancer cell lines (A549) were seeded using a density of 1 × 10^6^ in a 6-well culture plate. Subsequently, after 24 h, the medium was poured, and the cells were exposed to the treatment of curcumin, curcumin-loaded QCS-Vani imine nano-micelles and blank nano-micelles at the IC_50_ values and subjected to incubation in a 5% CO_2_ environment at 37 °C for 24 h. Following incubation, the cells were detached with the solution of trypsin–EDTA and again suspended in the freshly prepared medium. The cells were centrifuged (500× *g*, 5 min), leading to the formation of the cell pellets, which were again suspended using cold PBS of pH 7.4, followed by staining with annexin V-FITC (5 µL) and 3 µL PI, and incubation in a light-deprived chamber at room temperature for 15 min. Lastly, 100 µL of Annexin V binding buffer was poured, and the cells were analyzed through a flow cytometer with the collection of green fluorescence (FITC) at 535 nm and red (PI) fluorescence at 550 nm (1000 cells).

### 2.12. Cell Cycle Study

The effect of curcumin and QCS-Vani imine nano-micelles loaded with curcumin on the cell cycle was evaluated using flow cytometric analysis. For the analysis of the DNA contents, propidium iodide was used. About 1 × 10^6^ cells of A549 cells were cultured using 6-well culture plates. The cells were subjected to incubation for 24 h in the presence and absence of the IC_50_ concentration of curcumin and QCS-Vani imine nano-micelles loaded with curcumin. After incubation, the trypsinization of the cells was performed, which was followed by repeated washing with PBS and centrifugation (500× *g*, 5 min). After this, 10^6^ cells were collected and fixed in 70% ethanol at −20 °C overnight. The fixed cells were washed twice with PBS, and the cell pellet was re-suspended with 1 mL of cell cycle medium (100 µg/mL of RNase A, 50 µg/mL of PI, and 0.1% of Triton-X 100) and then further incubated at 30 °C in the dark for 30 min. Cells were analyzed using a flow cytometer with an excitation wavelength of 488 nm and an emission wavelength of 618 nm.

### 2.13. Evaluation of the Synergistic Effect of Cisplatin with Curcumin-Loaded QCS-Vani Imine

To evaluate the possible synergistic anticancer effect of cisplatin in combination with curcumin and curcumin-loaded QCS-Vani imine nano-micelles against lung cancer (A549) cell lines, the various concentrations of cisplatin were combined with the 5 µM curcumin and curcumin-loaded nano-micelles containing 5 µM of curcumin [36]. Different concentrations of cisplatin were applied in combination with a non-toxic concentration (IC_5_) of curcumin and QCS-Vani imine nano-micelles. The cytotoxicity evaluation was also performed in a similar pattern against mouse fibroblast L929 cell lines. The cell survival was assessed by the addition of 0.5 mg/mL MTT solution (100 µL). After 3 h, 100 µL of DMSO was added. The optical absorbance was measured at 550 nm. The synergistic anticancer effect was evaluated using a combination index (*CI*), which was calculated by using the below given equation

$$CI=\frac{C_{ax}}{I_{ca}}+\frac{C_{bx}}{I_{cb}}$$

*C_ax_* and *C_bx_* are the concentrations of drugs A and drug B used in combination to reach an IC_50_ value. *I_ca_* and *I_cb_* are the IC_50_ values for single A and B drugs.

### 2.14. Statistical Analysis

The experiments in this study were performed and the readings were collected in triplicate, which were represented as the average mean ± standard deviation. For the determination of statistical significance, “one-way ANOVA”, followed by a multiple comparison test, “Tukey”, was used.

## 3. Results and Discussion

### 3.1. Preparation and Characterization of QCS-Vani Imine Conjugate

**Figure 1:**
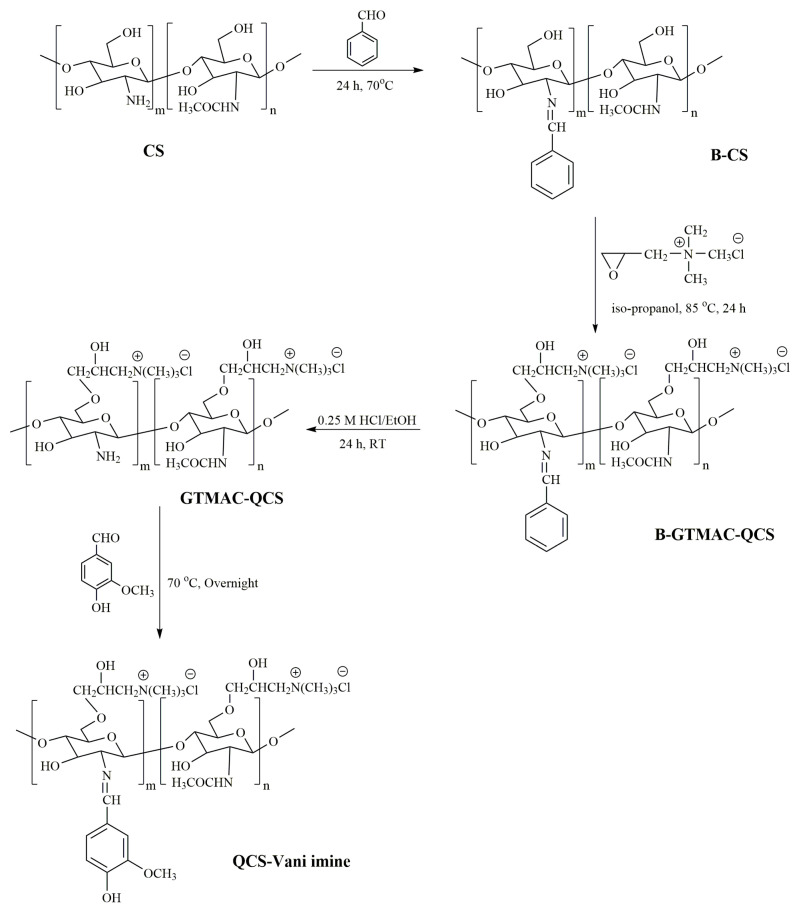
Synthesis route of vanillin–glycidyltrimethylammonium–quarternized chitosan (QCS-Vani imine) conjugate.

In this study, curcumin, a potent pharmacologically active compound, was loaded into the nano-micelles prepared through using a conjugate polymer system comprised of vanillin and quarternized chitosan, which was applied as a carrier. This system was employed for the very first time for the loading of curcumin and evaluated for its anticancer potential. The conjugate was prepared through the Schiff base chemical reaction. There are four steps involved in the synthesis of vanillin–glycidyltrimethylammonium–quarternized chitosan (QCS-Vani imine) conjugates, as shown in Figure 1. In the first step, benzyl–chitosan (B-CS) was formed by using the Schiff base chemical reaction of chitosan and benzaldehyde. In the second step, benzyl–glycidyltrimethylammonium–chitosan (B-GTMAC-QCS) was formed by the reaction of glycidyltrimethylammonium chloride (GTMAC) with B-CS. In the third step, GTMAC-QCS was formed by the de-protection of the amino groups of chitosan using 0.25 M HCl in EtOH. In the last step, QCS-Vani imine was synthesized by the Schiff base formation of GTMAC-QCS and vanillin. The final product prepared was subjected to chemical characterization to confirm its successful synthesis.

**Figure 2:**
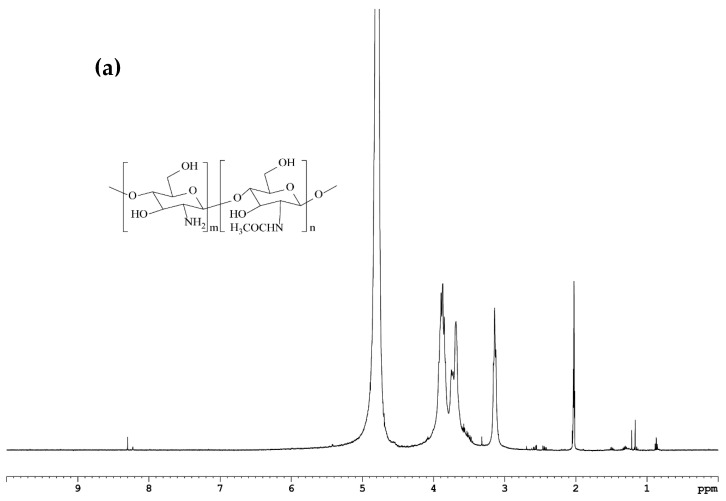

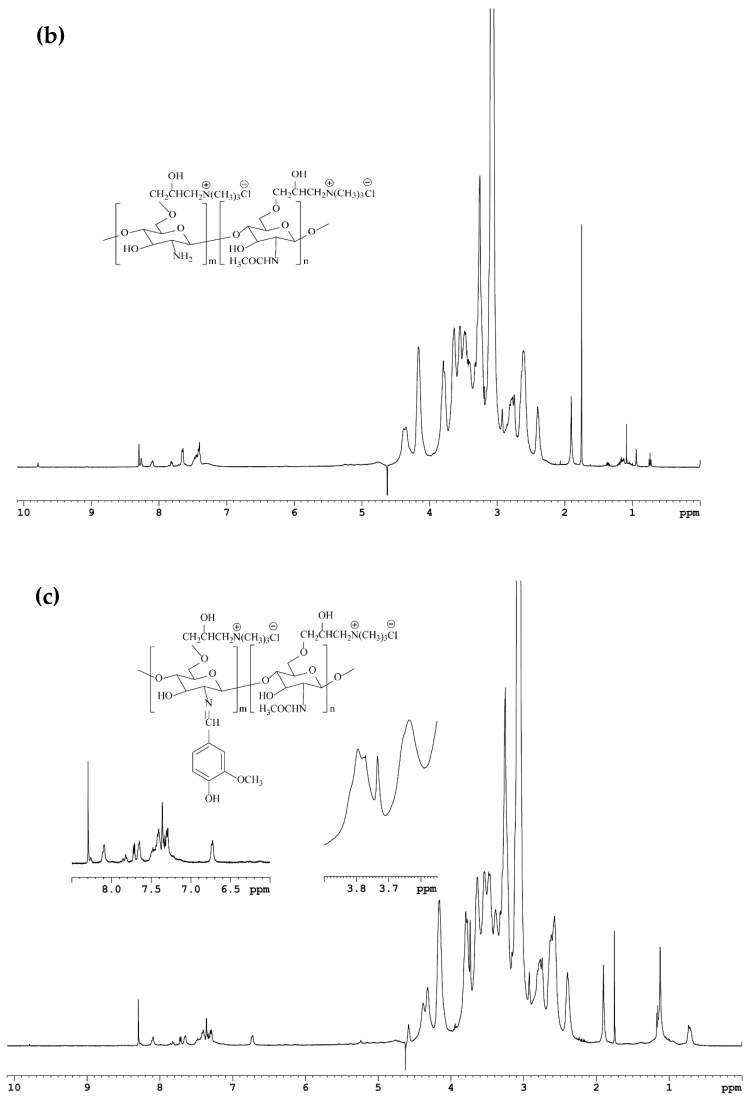
Spectra of ^1^H-NMR (500 mHz) of (**a**) chitosan (80 KDa, in deuterated methanol, spiked with D_2_O and acetic-D_3_. (**b**) GTMAC-CS in D_2_O and (**c**) QCS-Vani imine conjugate in D_2_O.

The ^1^H-NMR analysis was performed to confirm the formation of the QCS-Vani imine conjugate. The findings revealed the successful synthesis of the QCS-Vani imine conjugate as evident in Figure 2c in comparison to the spectra of chitosan (Figure 2a) and GTMAC-CS (Figure 2b). Results of ^1^H-NMR of QCS-Vani imine (Figure 2c) show signals at 7.4 to 8.0 ppm, which correspond to the aromatic proton of the attached vanillin, whereas the signals for aldehyde protons at 9.5 to 10.0 ppm disappeared. The signals of vanillin aromatic protons are missing in the spectrum of GTMAC-CS (Figure 2b), which indicates the attachment of vanillin to the chitosan backbone. Moreover, there is a signal at 8.3 ppm which corresponds to the imine proton indicating the successful formation of the imine bond between the chitosan and vanillin [37]. The findings of our study are in agreement with previous reports which have demonstrated the presence of aromatic proton signals at 7.4 to 8.0 ppm [28]. Moreover, the signal at 3.6 ppm in the spectrum of QCS-Vani imine was found absent when compared to the spectrum of GTMAC-CS, indicating the presence of protons of the aromatic *O*-methoxy group of vanillin [38]. These data support the successful substitution of vanillin on the QCS network and show the presence of vanillin in the final product. The ^1^ H-NMR analysis provides evidence for the substitution of vanillin but with a relatively low degree. The signals that emerged at 3.2 ppm in the ^1^H-NMR spectrum of GTMAC-CS are due to the protons of the quaternary group, which indicates the degree of quaternary substitution on chitosan. These data are in correlation with previous findings [28,39]. Overall, the findings support the successful formation of the desired product of the QCS-Vani imine conjugate.

**Figure 3:**
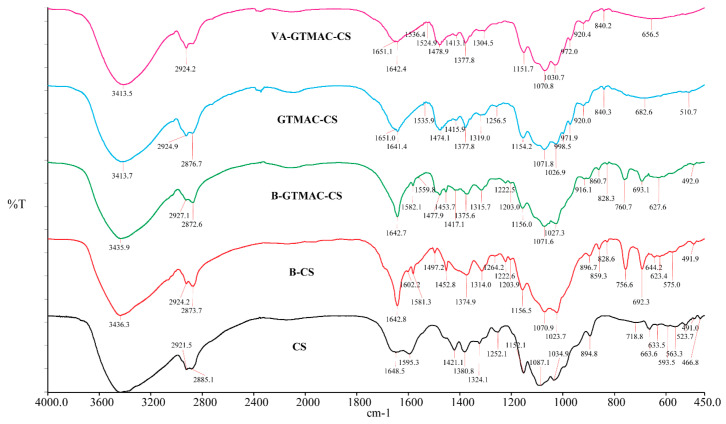
FT-IR of chitosan (CS), benzyl–chitosan (B-CS), benzyl–glycidyltrimethylammonium–chitosan (B-GTMAC-QCS), O-glycidyltrimethyl–ammonium chitosan (GTMAC-QCS), vanilin–glycidyltrimethylammonium–chitosan (QCS-Vani imine).

The FT-IR spectra of chitosan (CS), benzyl–chitosan (B-CS), benzyl–glycidyl trimethylammonium–chitosan (B-GTMAC-QCS), glycidyltrimethylammonium–chitosan (GTMAC-QCS) and vanillin–glycidyltrimethylammonium–chitosan (QCS-Vani imine) are shown in Figure 3. The FT-IR spectrum of CS showed a broadband at 3436.7 cm^−1^ attributed to -O-H and -N-H stretching. The peak at 1648.5 corresponds to the presence of amide-I, while the peak at 1595.3 cm^−1^ is attributed to the appearance of the amine (NH_2_) absorptions of CS, respectively. Some new peaks were observed in the FTIR spectra of B-CS when compared with CS, such as a peak at 1642.8 cm^−1^ which is attributed to the characteristic vibration of imine (C=N). The presence of a peak at 1640 cm^−1^ in GTMAC-CS corresponds to the carbonyl (C=O) of amide. Moreover, the presence of vanillin in the final product can be evidenced by the absence of an aldehyde peak, which usually emerges at 1600 cm^−1^ [40]. The absorption bands at 1581, 756.6 and 692.3 cm^−1^ correspond to the characteristic bending vibration of benzene. The FT-IR spectrum of B-GTMAC-QCS exhibited a sharp peak at 1477.8 cm^−1^ which corresponded to the characteristic stretching (asymmetrical) of the C-H bond in the methyl groups, which is one of the characteristics of the quaternary ammonium groups. After removing the benzyl group (Q-CS), the absorption peak at 1640.1 cm^−1^ resulting from the benzaldehyde group disappeared. The FT-IR spectrum of QCS-Vani imine showed a characteristic peak at 1642.6 cm^−1^ which corresponded to the C=N characteristic vibration of imines [27]. Similar spectra appeared at 1641.4 cm^−1^ in the FTIR spectra of GTMAC-CS, which corresponds to the carbonyl of amide [41]. The pattern of these two spectra is different, which indicates a difference in the functional moieties. This provides evidence for the presence of vanillin attachments to the chitosan network. Similarly, the yield of QCS-Vani imine was found to be 93.18%, and the degree of quaternary ammonium (DQ) on GTMAC-QCS was 33%. The degree of substitution (DS) of vanillin on QCS-VANI IMINE was found to be 3.02 ± 0.13%.

**Figure 4:**
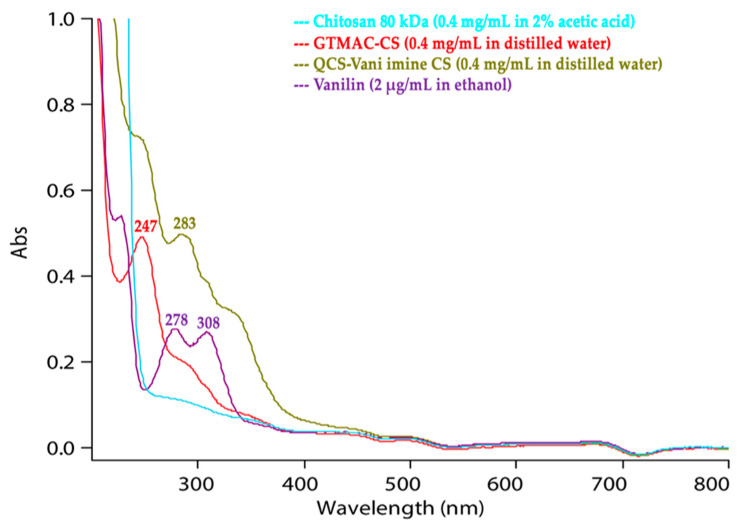
UV visible spectra of chitosan 80 kDa in 2% acetic acid, GTMAC-CS in distilled water, QCS-Vani imine in distilled water and vanillin in ethanol.

The UV-visible spectra of chitosan, GTMAC, QCS-Vani imine and free vanillin are given in Figure 4. Due to the absence of chromophores in the structure of chitosan, there was no clear absorption peak observed in the UV spectrum. The vanillin structure contains a carbonyl group and a benzene ring, which caused a strong absorption peak in the 278–308 nm range of wavelength. Due to the effect of the polarity of the solvent, the maximum absorption wavelength of the final product, i.e., QCS-Vani imine, shifted. The free vanillin sample was prepared using ethanol, whereas the QCS-Vani imine sample was prepared in distilled water. The selection of the solvent was based on the polarity of the respective solvent. The shift in the peak of the QCS-Vani imine might be due to the effect of the solvent. The various solvents have different pH values, and it is well demonstrated that pH markedly causes a shift of the peak. In the current study, the slight shift in the peak of vanillin in the QCS-Vani imine conjugate might be due to the slightly lower pH of water compared to ethanol [41]. Moreover, there is evidence that has demonstrated the shift of absorption peaks of vanillin and chitosan conjugation due to a lowering in the energy of the system, which results in the redshift [42]. Overall, the findings support the presence of vanillin in the final product.

### 3.2. Curcumin-Loaded QCS-Vani Imine Nano-Micelles

**Figure 5:**
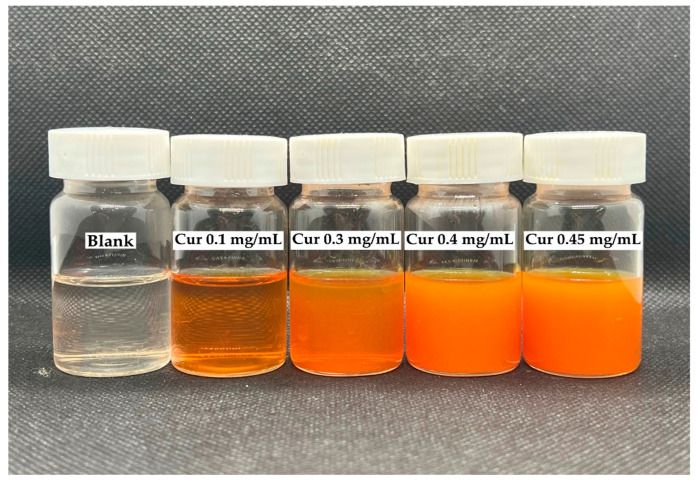
Pictures of curcumin-loaded QCS-Vani imine micelles using different concentrations (0.1, 0.3, 0.4 and 0.45 mg/mL) of curcumin in the *QCS-Vani imine* (3 mg/mL) nano-micelles preparation. Blank is nano-micelles of 3 mg/mL *QCS-Vani imine* without curcumin.

QCS-Vani imine nano-micelles were successfully loaded with curcumin. The loaded nano-micelles products were obtained as presented in Figure 5. The resulting QCS-Vani imine nano-micelles loaded with curcumin were obtained as clear dark orange–yellow solutions. In the experiment, when curcumin of more than 0.45 mg/mL was added to the QCS-Vani imine solution (3 mg/mL), precipitation was observed. The precipitation occurred at the dialysis stage due to the over-maximum loading capacity of the QCS-Vani imine nano-micelles. Therefore, the optimum concentration of curcumin was found to be 0.45 mg/mL. To remove excess uncapped curcumin, the dialysis method was utilized. The solutions of curcumin-loaded QCS-Vani imine nano-micelles were prepared in 90% DMSO, kept in a dialysis bag (MW cut-off 3500), and subjected to the process of dialysis using distilled water. The excess curcumin was released slowly and steadily, which consequently allowed the entry of water into the dialysis bag in an equilibrium manner. The very steady and slow influx of water and outflux of DMSO into the dialysis bag resulted in the removal of excess curcumin and thus prevented the precipitation of curcumin. Stable micelle formation was observed at a concentration of 0.45 mg/mL of curcumin, which was marked as the optimum concentration. Our findings are in agreement with previous reports [40,43].

### 3.3. Characterization of Curcumin-Loaded QCS-Vani Imine Nano-Micelles

#### 3.3.1. CMC of QCS-Vani Imine Nano-Micelles

**Figure 6:**
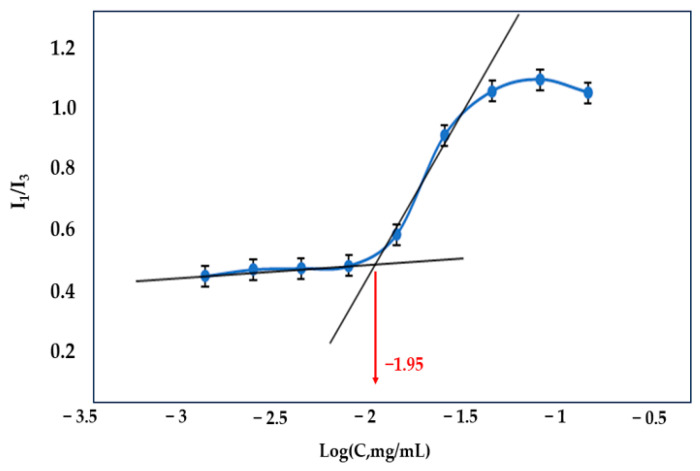
Critical micelle concentration (CMC) determination of QCS-Vani imine. All the values are represented as mean ± standard deviation (n = 3).

The CMC of nano-micelles can be described as the minimum concentration of polymers that is sufficient for the formation of micellar aggregations. The CMC value of a copolymer indicates a thermodynamic feature that pertains to its self-assembly characteristics in an aqueous medium. It works as well-demonstrated and acceptable criteria for evaluating the thermodynamic stability of the nano-micelles [33]. In this study, the CMC was assessed by monitoring the change in the intensity ratio of I_1_/I_3_ (I_1_ at 375 nm and I_3_ at 390 nm) of pyrene fluorescence emission spectra at different concentrations of QCS-Vani imine. The results shown in Figure 6 demonstrated that the CMC value for QCS-Vani imine was 0.012 mg/mL.

#### 3.3.2. Particle Size Analysis, Zeta Potential and Polydispersity Index (PDI)

Table 1 displays information on the sizes of the QCS-Vani imine nano-micelles loaded with curcumin in various concentrations. The findings indicated that the size of curcumin-loaded nano-micelles with varying concentrations of curcumin was the same. All the formulations showed particle sizes ranging from 208 to 283 nm. The size of nano-micelles reported in our study is considered suitable for the delivery of therapeutic agents with anticancer properties [44]. The zeta potential of all the curcumin-loaded QCS-Vani imine nano-micelles was around +30 mV, as shown in Table 1. This was pretty much the same as the zeta potential of the blank QCS-Vani imine nano-micelles. It is therefore presented that the positive charge outside nano-micelles was not disturbed by the drug entrapment. All samples with a PDI less than 0.25 display a narrow distribution. The curcumin-loaded QCS-Vani imine nano-micelles were properly powdered using the freeze-drying technique and glycine as a cryoprotectant. The size of curcumin-loaded QCS-Vani imine micelles for re-dispersion of the nano-micelle powder in water was found to be 218.61 ± 2.12 nm, with a polydispersity index value of less than 0.184, showing a uniform distribution of particles in the formulation and indicating the stability of the powdered form the nano-formulation.

#### 3.3.3. Percent Entrapment Efficiency (%EE) and Drug-Loading Capacity

The percent entrapment efficiency (%EE) and percent drug loading capacity (%LC) of QCS-Vani imine nano-micelles loaded with curcumin were determined by HPLC. The concentration of the polymer conjugate was 3 mg/mL, and various concentrations of curcumin were used in this study. The results demonstrated the highest %EE (67.61%) of the nano-micelles when curcumin was used at a concentration of 0.3 mg/mL (Table 2). However, increasing the concentration of curcumin to 0.45 mg/mL resulted in a much lower %EE, which may be due to reaching the maximum loading capacity as well as the lower water solubility of curcumin, which could have resulted in lower entrapment efficacy [44]. Similarly, the maximum drug-loading capacity (6.15 ± 0.41) was observed at a 0.3 mg/mL concentration of curcumin (Table 2). Therefore, the optimum concentration of curcumin, which has shown the highest %EE and %LC in the nano-micelles, was proceeded further in the experiments.

#### 3.3.4. Morphological Study Using Transmission Electron Microscopy (TEM)

**Figure 7:**
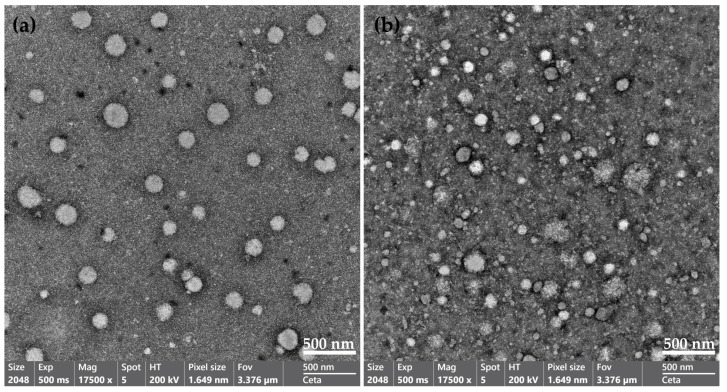
TEM micrographs of (**a**) blank nano-micelles and (**b**) curcumin-loaded QCS-Vani imine nano-micelles at 17,500 magnifications.

Morphological characterization of the nano-micelles using TEM analysis was carried out for blank (unloaded nano-micelles) and curcumin-loaded QCS-Vani imine nano-micelles at 17,500 magnification, which showed the nano-micelles in a spherical shape (Figure 7). The diameter of blank and curcumin-loaded QCS-Vani imine micelles was about 200 nm and 150 nm, respectively. The variations observed in the sizes of the unloaded and loaded nano-micelles might be due to the interaction of curcumin and vanillin through π–π interaction. Vanillin molecules constitute the inner walls of the spherical particles in QCS-Vani imine nano-micelles. Once curcumin molecules are loaded, vanillin molecules form a π–π aromatic linkage with the aromatic moieties of curcumin, resulting in a shrinkage of the particle sizes and, consequently, a reduction in the sizes of the nano-micelles, as can be observed in Figure 7. The smaller size of curcumin-loaded nano-micelles observed can also be attributed to the nano-ionization of curcumin, which helps with the improvement of the bioavailability of hydrophobic drugs. The results of our data agree with previously reported data in the literature [40].

### 3.4. Stability Study of the Curcumin-Loaded Loaded QCS-Vani Imine Nano-Micelles Powders

The stability of the freeze-dried powdered form of curcumin-loaded QCS-Vani imine nano-micelles was assessed at two different temperatures with a storage duration of up to 120 days. The stability of the stored samples was evaluated by an assessment of the changes in their particle sizes, values of zeta potential and percent remaining of the loaded curcumin contents. Curcumin-loaded QCS-Vani imine nano-micelles stored at 4 °C were found to have better stability than those stored at 30 °C. At day 0, the sizes of curcumin-loaded nano-micelles were 264.87 ± 4.51 and were found to slightly increase during 16 weeks of storage time at 4 °C (303.11 ± 3.76, at week 16). In contrast, the nano-micelles stored at 30 °C experienced a dramatic increase in their particle size during 16 weeks of storage. The samples were stable during the first 14 days, and at the end of the 16th week, their size increased to 506.94 ± 3.22. The findings revealed the samples stored at 4 °C presented higher stability by showing consistency in their size compared to the samples that were stored at 30 °C. The stability of the nano-micelle powder was also evaluated by measuring the percentage of the remaining contents of curcumin encapsulated by storing the samples at 4 °C and 30 °C for 120 days. The findings revealed that the QCS-Vani imine nano-micelles loaded with curcumin showed higher stability at 4 °C compared to the samples stored at 30 °C by showing high contents of encapsulated curcumin. The curcumin contents of the samples stored at 30 °C were found stable only during the first 14 days; then, they reduced afterward. The curcumin contents of the samples stored at 4 °C were found stable throughout the 120 days of storage time compared to day 0. It is therefore suggested that curcumin-loaded QCS-Vani imine nano-micelle powders should be kept in a refrigerator (4 °C) and protected from light to maintain the stability of the encapsulated drug.

**Figure 8:**
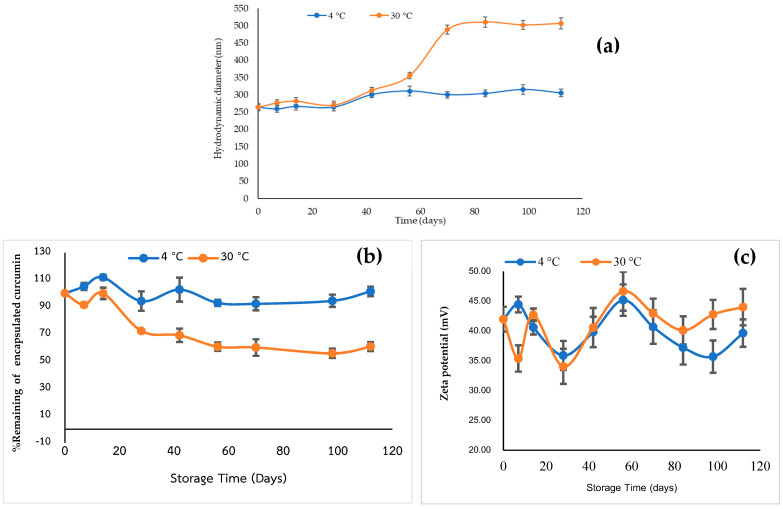
Stability study of curcumin-loaded QCS-Vani imine nano-micelles powders stored at 4 °C and 30 °C for 120 days: (**a**) particle size, (**b**) the % remaining of curcumin contents, and (**c**) the zeta potential (mV). The values are represented as mean ± standard deviation where (n = 3).

The values of the zeta potential of the QCS-Vani imine nano-micelles loaded with curcumin are presented in Figure 8c. The samples showed no statistically significant difference in the values of the zeta potential during the 120-day storage period, which was found to be in the range of +34 to +46 mV.

### 3.5. DSC Analysis

**Figure 9:**
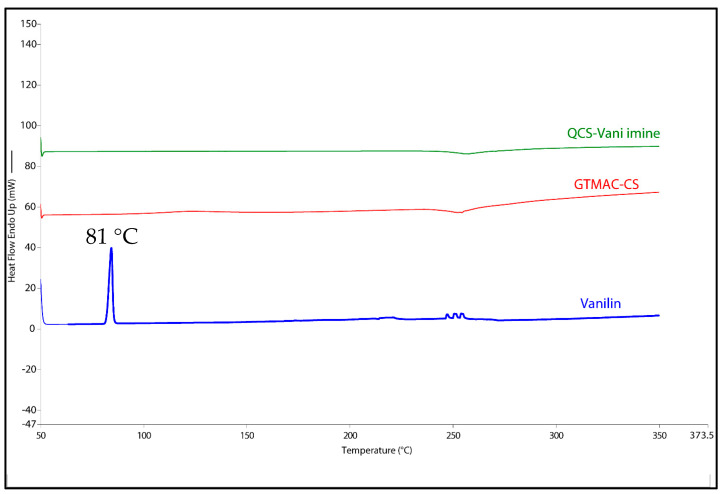
DSC thermograms of vanillin, GTMAC-CS and QCS-Vani imine.

The DSC analysis was performed to find out if there was any free vanillin in the final product of the QCS-Vani imine conjugate. The excess of free vanillin was completely removed using the dialysis method. Figure 9 shows the melting endothermic peak of vanillin (at 81 °C) disappearing in the final product. This indicates that the final product of the QCS-Vani imine was successfully obtained with no free vanillin remaining.

### 3.6. In Vitro Drug Release

**Figure 10:**
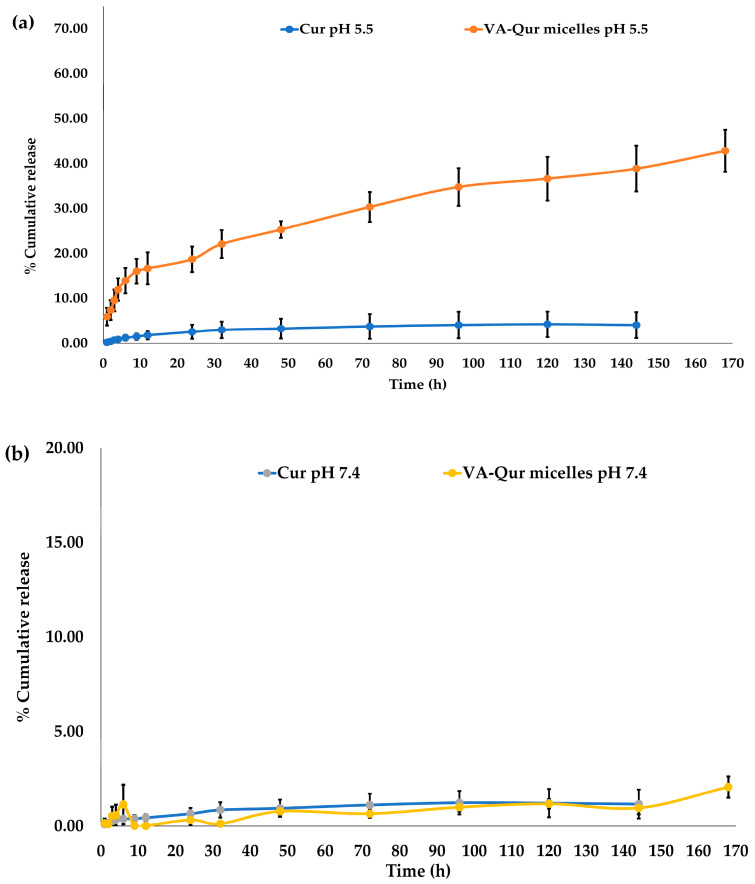
The release profiles of curcumin from curcumin micelles and curcumin-loaded QCS-Vani imine nano-micelles in phosphate buffer having pH 5.5 (**a**) and 7.4 (**b**) containing 0.5% tween-80.

An in vitro drug release study was performed using the dialysis method to study the curcumin release behavior of the developed system. The release profile of curcumin from the loaded QCS-Vani imine nano-micelles and pure curcumin in phosphate buffer of pH 7.4 (representing blood) and phosphate buffer of pH 5.5 (representing the tumor environment) was evaluated at 37 °C, and the findings are given in Figure 10. In phosphate buffers of pH 5.5 and 7.4, drug release of the pure curcumin was very low (~4.0% (pH 5.5), ~1.2% (pH 7.4)) after 144 h. Similarly, drug release from the curcumin-loaded QCS-Vani imine nano-micelles in phosphate buffer pH 7.4 showed the same pattern as shown by pure curcumin (~1.2%). However, the release profile of curcumin-loaded QCS-Vani imine nano-micelles in phosphate buffer of pH 5.5 showed sustained release, i.e., 40% after 144 h. The results revealed that curcumin-loaded QCS-Vani imine nano-micelles have pH-responsive behavior. Actually, the presence of a quarternized ammonium group on the chitosan tends to affect the behavior of the nano-micelles toward pH responsiveness. It has been observed that nano-micelles loaded with chitosan show pH-responsive behavior in water and physiological fluids. At low pH, it forms a vicious solution, while at high pH, it becomes insoluble. It happens due to the protonation and deprotonation processes of the amine groups on the chitosan polymer chain. The quarternized chitosan is more susceptible to such a process due to the presence of a quaternary ammonium group. For the pH-responsive QCS-Vani imine nano-micelles system, the curcumin release rate was significantly increased with the decrease in pH value [31]. It is well demonstrated that a typical tumor has a microenvironment with an acidic pH which is attributed to its acidic metabolic waste products accumulating in the tumor. These environmental characteristics of a tumor may offer physicochemical features that are ambient for developing selective tumor recognition therapy. The developed delivery system is shown to have pH-responsive properties. In lower pH environments, the anticancer agent curcumin was observed with a better release from the nano-micelles than in blood circulation. This will provide more selectivity toward cancer cells than normal cells. Moreover, the system could prevent release of the encapsulated drug in the blood circulation system and deliver it to the target cancer without releasing the active drug during circulation.

### 3.7. Anticancer Evaluation

Curcumin is a well-established natural compound with promising anticancer effects reported in various investigations. However, the compound has some limitations, including water solubility, that hinder its way to clinical trials and anticancer use in humans. Nano-technological approaches offer the solution for such constraints and provide better outcomes [45,46]. In this study, a conjugated polymer system was applied for the preparation of nano-micelles and loaded with curcumin to increase the water solubility and consequently the anticancer activity of curcumin. The anticancer potential of curcumin and curcumin-loaded QCS-Vani imine nano-micelles was investigated against human lung carcinoma cell lines (A549), and their cytotoxicity was evaluated in rat embryonic cardio-myocytes (H9C2). The cytotoxicity efficiency was expressed in IC_50_ values, as summarized in Table 3 and Figures S2 and S3. The findings showed the cytotoxic nature of pure curcumin against all the cell lines that were evaluated in this study. However, after the encapsulation of curcumin in the developed nano-micelles, the cytotoxicity against cancerous cell lines was significantly improved. The lower IC_50_ values of all tested nano-micelles compared to pure compounds against cancer cell lines of about 2.68-folds were illustrated. Curcumin-loaded QCS-Vani imine nano-micelles improved selectivity from 0.37 to 1.60 in lung cancer A549 and normal H9C2 cell lines. From the cytotoxicity assay, it is obvious that the incorporation of curcumin in QCS-Vani imine nano-micelles could significantly increase anticancer activity and reduce toxicity toward normal cells compared with pure curcumin.

### 3.8. Cellular Uptake

**Figure 11:**
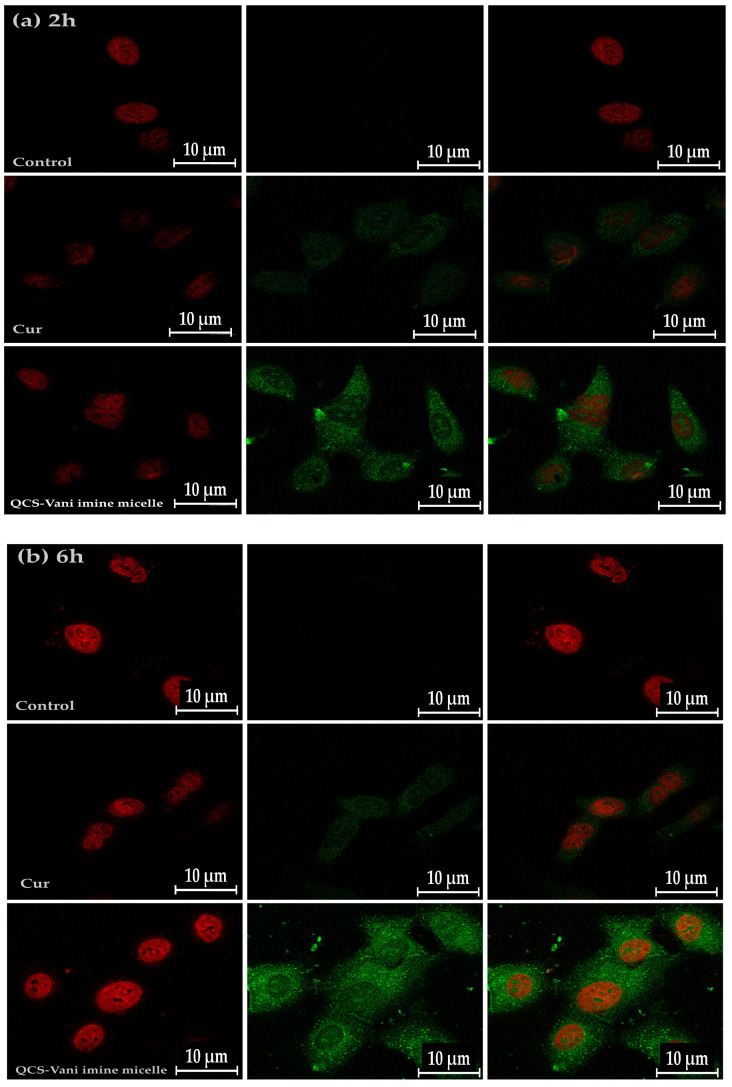

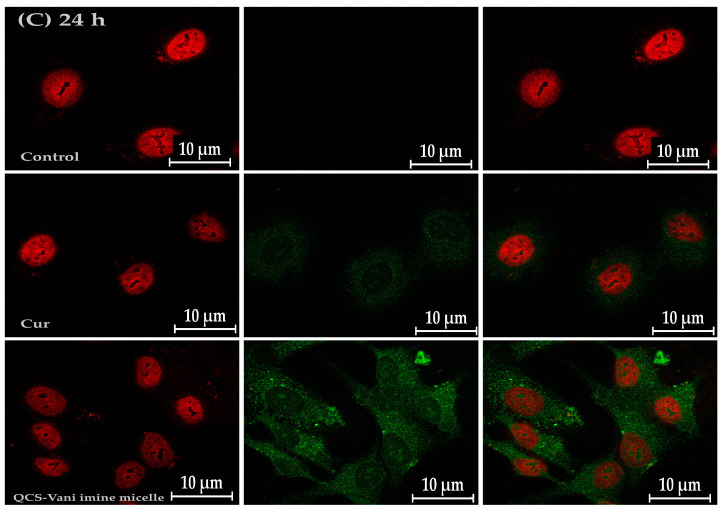
Confocal microscope images representing the cellular uptake of curcumin by lung cancer cell lines (A549). The calls and the samples were incubated for (**a**) 2 h, (**b**) 6 h, and (**c**) 24 h. The images show cell nucleus stained by Hoechst 33342 dye (red) and curcumin (green). The images were recorded at magnification 63×, scale bar: 10 μm.

The cellular uptake study of pure curcumin and QCS-Vani imine nano-micelles loaded with curcumin by the human lung cancer cell lines (A549) was carried out by using an imaging technique. Figure 11 shows the uptake of pure curcumin and curcumin-loaded QCS-Vani imine nano-micelles by A549 cells. The green color presents the curcumin distribution, while the red color shows the cell nucleus. Generally, Hoechst 33342 dye-stained cellular nuclei are usually blue in color; however, to prevent the contrast of the green and blue colors, the color of the nuclei was adjusted to red for clear visibility and convenience of the readers. The result showed that after 2 h of incubation, the green color of curcumin was observed in the cytoplasm. After 24 h, a greater amount of curcumin was detected in the cytoplasm, which is closely located near the nucleus.

Interestingly, curcumin-loaded QCS-Vani imine nano-micelles showed better uptake by A549 cells than pure curcumin at 2, 6, and 24 h. It has been previously observed that the amine moieties of chitosan in QCS-Vani imine nano-micelles become protonated in the extracellular environment of cancer cells (pH: approx. 6.8) and attached to the negatively charged cell membranes of cancer cells through an electrostatic force of attraction, and they enter the cells through endocytosis. The intracellular pH of cancerous cells is about 5.5, and the curcumin from QCS-Vani imine nano-micelles is released due to the breakage of the imine bond at this pH [40]. Moreover, it has been reported that chitosan opens the tight junctions in the cell membrane, which consequently facilitates the entry of the nano-micelles and ultimately the release of curcumin at pH 5.5 [47]. Therefore, increased uptake of curcumin by cancer cell lines was observed as compared to pure curcumin, because the poor water solubility of pure curcumin hinders its bioavailability and ultimately its uptake and anticancer effect [48]. The fluorescence intensities of curcumin and curcumin-loaded QCS-Vani imine nano-micelles measured by CSLM show relatively higher intensities at 6 h (Table 4). Overall, the findings support the conclusion that curcumin micelles have more cytotoxic activity toward lung cancer A549 cell lines compared to pure curcumin [49,50].

### 3.9. Evaluation of the Effect of QCS-Vani Imine on Cell Apoptosis

**Figure 12:**
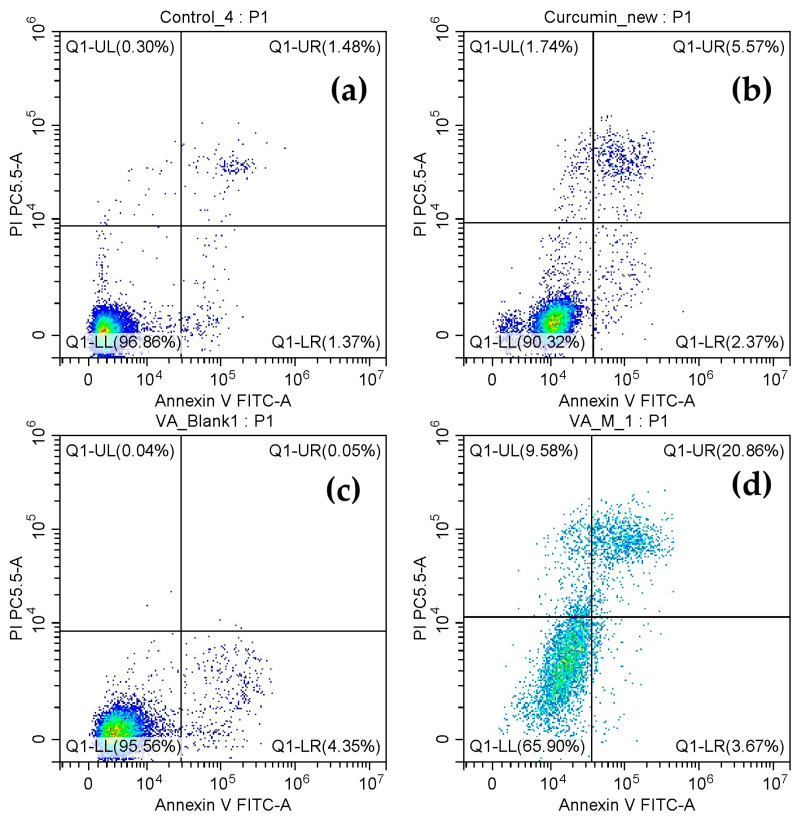
The effect of curcumin or curcumin-loaded QCS-Vani imine induced apoptosis on A549 cells: (**a**) control = no treatment, (**b**) pure curcumin, (**c**) blank QCS-Vani imine nano-micelles, (**d**) curcumin loaded QCS-Vani imine nano-micelles.

Apoptosis is a naturally occurring genetically programmed process during embryonic development [51]. The induction of apoptosis in A549 cells when treated with curcumin and curcumin-loaded QCS-Vani imine nano-micelles was studied by a flow cytometric technique. During the process of apoptosis, the cells transmit signals like phosphatidylserine (PS) to the phagocytic cells through the extracellular membrane with a message of clearance of the apoptotic cells. Annexin-V specifically attaches to the PS present in apoptotic cells, indicating the early stage of apoptosis. Necrosis or late-stage apoptosis may happen during this process. Annexin-V attaches to PS present on apoptotic cells, while PI stains the nucleus as a consequence of the loss of membrane integrity [52,53]. To delve deeper into the death pattern, the cells were treated for 24 h with curcumin-loaded QCS-Vani imine nano-micelles compared with pure curcumin. Apoptosis was detected by Annexin V-Fit C and PI analyses. For differentiation between necrosis and apoptosis, we conducted double staining using Annexin V-Fit C and PI, which was followed by flow cytometry. The cells that are viable do not attach to PI or Annexin V-Fit C (lower left quadrant); cells of early-stage apoptosis attach to Annexin V-Fit C but exclude PI (lower right quadrant); and cells of late-stage apoptosis or necrosis were positive for both Annexin V-Fit C and PI (upper right quadrant) (Figure 12).

The curcumin and curcumin-loaded QCS-Vani imine nano-micelles show their effect by inducing apoptosis in A549 cells. The results demonstrated that curcumin-loaded QCS-Vani imine nano-micelles showed a much better induction of apoptosis in A549 cells. The early apoptotic cells (Annexin V-Fit C positive and PI negative) represented 1.26% of the total A549 cells that curcumin was tested on. The late apoptotic cells (Annexin V-Fit C and PI positive) represented 7.26% of the total A549 cells that curcumin was tested on. The early apoptotic cell represented 4.71% of the total A549 cells that QCS-Vani imine nano-micelles loaded with curcumin were tested on. The late apoptotic cell represented 19.81% of the total A549 cells that QCS-Vani imine nano-micelles loaded with curcumin were tested on, as shown in Figure 12. Overall, curcumin-loaded QCS-Vani imine nano-micelles could enhance apoptosis induction in A549 cells and, therefore, may increase the cytotoxicity toward the lung cancer cells.

### 3.10. Evaluation of Effect of QCS-Vani Imine on Cell Cycle

**Figure 13:**
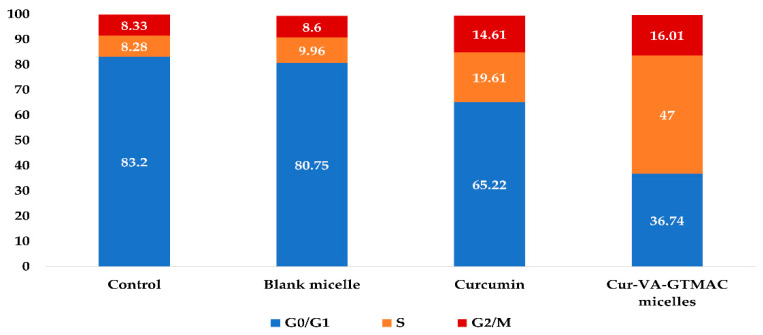
Cell cycle distribution of A549 cells treated by different samples.

The evaluation of the cell cycle of A549 cells treated with different IC_50_ concentrations of curcumin, blank nano-micelles and curcumin-loaded QCS-Vani imine nano-micelles was studied using flow cytometric analysis. A549 cells were treated with samples for 24 h, and then propidium iodide staining was utilized for the analysis of DNA content. The findings of the study revealed that blank nano-micelles and control-treated groups showed similar data after exposure to IC_50_ concentrations of the samples (Figure 13).

Curcumin produced the effect of cell cycle arrest at the S phase with a profound increase in the percentage from 8.28% to 19.61% of the cells at the S phase in comparison to the control. Interestingly, the percentages of cells in the S phase were markedly increased from 19.61% to 47% in groups treated with loaded QCS-Vani imine nano-micelles, showing a significant ability of the QCS-Vani imine nano-micelles of induction of cell cycle arrest in the S phase in comparison to pure curcumin (Figure 13). The cells will not proceed with mitosis if DNA replication is not complete.

### 3.11. Evaluation of Synergistic Effect of Cisplatin in Combination with Curcumin and Curcumin-Loaded QCS-Vani Imine Nano-Micelles

**Figure 14:**
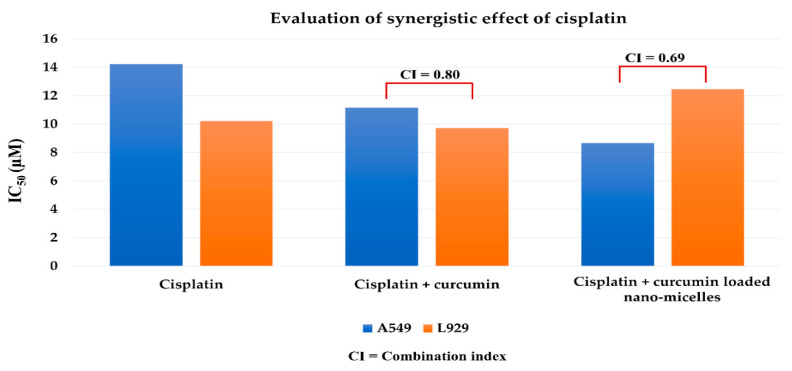
Synergistic anticancer effect of cisplatin with curcumin and curcumin-loaded nano-micelles.

Treatment of chronic pathological conditions often uses a combination of therapeutic agents that have significant therapeutic outcomes, particularly in the case of lethal diseases with an incidence of drug resistance [54]. Cisplatin is one of the most widely used chemotherapeutic agents, but the development of resistance and toxic effects on healthy cells hinder its applications. Therefore, cisplatin was combined in various concentrations with curcumin and curcumin-loaded QCS-Vani imine nano-micelles (Table S1) to evaluate the synergistic anticancer activity with no or minimum side effects on healthy cells. Curcumin and cisplatin share interesting chemistry and pharmacodynamic properties. It has been reported that curcumin reverses the resistance to cisplatin in lung cancer cell lines through various mechanisms. The p-glycoprotein inhibition capability of curcumin paves the way for the entry of cisplatin into cancer cells. Once cisplatin enters the cells, its efflux from the cells becomes difficult, and consequently, the availability of cisplatin in high concentrations will have a high anticancer effect [55]. Therefore, in this study, we studied the synergistic effect of cisplatin and curcumin-loaded nano-micelles. The findings revealed that cisplatin alone displayed anticancer activity against lung cancer cell lines with an IC_50_ value of 14.22 ± 1.01 µM, whereas the combination with curcumin gave a lower IC_50_ value of 11.15 ± 0.98 µM, and significantly higher activity was observed when combined with curcumin-loaded QCS-Vani imine nano-micelles (IC_50_ = 8.66 ± 0.88 µM), as shown in Figure 14 and Figure S4. Overall, the findings encourage the use of cisplatin in combination with curcumin and curcumin-loaded nano-micelles for enhanced anticancer activity. Not only the synergistic effect in anticancer activity when using cisplatin with curcumin-loaded nano-micelles but also less toxicity to normal cells was observed, as shown in Figure 14 and Figure S5. Significantly higher IC_50_ values of cisplatin against L929 were observed in comparison with treatment with cisplatin alone or cisplatin in combination with curcumin. This result demonstrated that the developed system could improve the selectivity of the cancer cell to normal cells, leading to fewer adverse effects of using an anticancer agent such as cisplatin in this study.

## 4. Conclusions

This study provides a deeper insight into the application of quarternized chitosan conjugated with vanillin imine as a carrier for anticancer agents. The conjugate was successfully prepared through a Schiff base formation and was well characterized. Curcumin was loaded in QCS-Vani imine nano-micelles. The nano-micelles were obtained in less than 218 nm size in a spherical shape, and a high %EE was obtained up to 67.61%. The QCS-Vani imine nano-micelles containing curcumin show different release patterns in different pH mediums. From the result, it was found that curcumin-loaded QCS-Vani imine nano-micelles have pH-responsive behavior. They could release the drug well at pH = 5.5 and slowly release it at pH = 7.4. Curcumin-loaded QCS-Vani imine nano-micelles showed high selectivity to lung cancer cells, lower toxicity to the normal cells and higher activity than pure curcumin. Curcumin-loaded QCS-Vani imine nano-micelles enhanced cell cycle arrest at the S phase against A549 cells, and the curcumin micelles showed much better-induced apoptosis compared with pure curcumin, which could improve the anti-lung cancer activity of curcumin. Moreover, cisplatin showed synergistic anticancer effects with curcumin and curcumin-loaded QCS-Vani imine nano-micelles against lung cancer cell lines with low toxicity to normal cells. From the results, it can be concluded that the developed QCS-Vani imine nano-micelles drug delivery system could improve the lung anticancer activity of anticancer drugs and could be a promising approach for treating lung cancer. However, further research is needed at acute and sub-acute levels on the toxicity aspects of the developed vehicle toward blood cells and vital organs.

## Figures and Tables

**Table 1:** Details of concentrations and volumes of QCS-Vani imine and curcumin used in nano-micelles preparation.

Sample	The Volume of 4 mg/mL QCS-Vani Imine Solution (mL)	Volume of 10 mg/mL Curcumin (mL)	The Volume of DMSO (mL)	Amount of Curcumin (mg)
Blank micelles	7.5	0	2.5	0
Curcumin 0.1 mg/mL	7.5	0.1	2.4	1
Curcumin 0.3 mg/mL	7.5	0.3	2.2	3
Curcumin 0.4 mg/mL	7.5	0.4	2.1	4
Curcumin 0.45 mg/mL	7.5	0.45	2.05	4.5

QCS-Vani imine = quarternized chitosan–vanillin; DMSO =dimethyl-sulfoxide.

**Table 2:** Particle size, zeta potential, polydispersity index (PDI) value, %entrapment efficiency and drug-loading capacity of curcumin-loaded QCS-Vani imine (3 mg/mL) prepared by dialysis method with various concentrations of loaded curcumin.

Curcumin (mg/mL)	Size (nm)	Zeta Potential (mV)	PDI	%Entrapment Efficiency	%Drug-Loading Capacity
0.1	283.15 ± 5.13	36.95 ± 1.77	0.232 ± 0.01	42.36 ± 2.57	1.32 ± 0.07
0.3	218.61 ± 2.12	37.35 ± 1.20	0.184 ± 0.01	67.61 ± 5.00	6.15 ± 0.41
0.4	208.65 ± 2.33	38.40 ± 1.56	0.181 ± 0.02	40.64 ± 7.30	4.78 ± 0.77
0.45	219.55 ± 4.74	38.35 ± 1.20	0.245 ± 0.04	40.39 ± 2.32	5.27 ± 0.27

All the values are represented as mean ± standard deviation where (n = 3).

**Table 3:** The IC_50_ values and selectivity of curcumin and curcumin-loaded QCS-Vani imine nano-micelles against A549 and H9C2 cell lines.

Sample	A549 IC_50_ (µM)	H9C2 IC_50_ (µM)	Selectivity (Fold)
Curcumin	86.69 ± 4.45	32.32 ± 2.34	0.37
Curcumin-loaded QCS-Vani imine nano-micelles	32.84 ± 2.6	52.44 ± 3.78	1.60

The values are represented as mean ± standard deviation, where (n = 3). A549 cells: lung carcinoma cell line, H9C2 cells: rat embryonic cardio-myocyte cell line.

**Table 4:** Fluorescence intensity of curcumin and curcumin-loaded QCS-Vani imine during cell uptake study measured by CSLM.

Sample	Time (h)	Fluorescence Intensity
Curcumin	2	0.629
Cur QCS-Vani imine nano-micelles	2	5.127
Curcumin	6	1.198
Cur QCS-Vani imine nano-micelles	6	6.998
Curcumin	24	0.893
Cur QCS-Vani imine nano-micelles	24	5.96

## Data Availability

The data presented in this study are available on request from the corresponding author.

## Supplementary Materials

The following are available online at https://www.mdpi.com/article/10.3390/jfb14100525/s1, Figure S1: Chemical structure of curcumin. Figure S2: Cell viability of human non-small cell lung cancer cell line (A549) after 24 h treatment with various concentrations of curcumin and curcumin loaded QCS-Vani imine nano-micelles. All the values are expressed as mean ± standard deviation (n = 3) of curcumin concentrations. Figure S3: Cell viability of rat cardiomyocytes cell line (H9C2) after 24 h treatment with various concentrations of curcumin and curcumin loaded QCS-Vani imine nano-micelles. All the values are expressed as mean ± standard deviation (n = 3) of curcumin concentrations. Figure S4: Cell viability of human non-small cell lung cancer cell line (A549) after 24 h treatment with various concentrations of cisplatin (in blue), cisplatin co-treatment with curcumin at 5 µM (IC_5_) (in orange), cisplatin co-treatment with curcumin loaded QCS-Vani imine at 5 µM (IC_5_) (in grey). All the values are expressed as mean ± standard deviation (n = 3) of cisplatin concentrations (µM). Figure S5: Cell viability of mouse fibroblast cell line (L929) after 24 h treatment with various concentrations of cisplatin (in blue), cisplatin co-treatment with curcumin at 5 µM (IC_5_) (in orange), cisplatin co-treatment with curcumin loaded QCS-Vani imine at 5 µM (IC_5_) (in grey). All the values are expressed as mean ± standard deviation (n = 3) of cisplatin concentrations (µM). Table S1. Concentration ratios of cisplatin to curcumin when various concentrations of cisplatin co-treatment with curcumin at 5 µM (IC_5_) and with curcumin loaded QCS-Vani imine at 5 µM (IC_5_).
