# Supplementary material for: Preparation of Self-Assembled, Curcumin-Loaded Nano-Micelles Using Quarternized Chitosan–Vanillin Imine (QCS-Vani Imine) Conjugate and Evaluation of Synergistic Anticancer Effect with Cisplatin
Source: J Funct Biomater. 2023 Oct 18;14(10):525. doi: 10.3390/jfb14100525 (PMC10607333; doi:10.3390/jfb14100525)
Supplement: Supplementary file 1 [file jfb-14-00525-s001.zip › jfb-2629640-supplementary.pdf]

Supplementary materials

Preparation of self-assembled curcumin loaded nano-micelles using quarternized chitosan-vanillin imine (QCS-Vani imine) conjugate and evaluation of synergistic anticancer effect with Cisplatin

Figures:

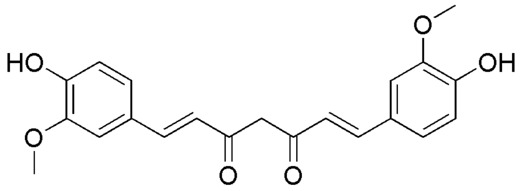

Figure S1: Chemical structure of curcumin.

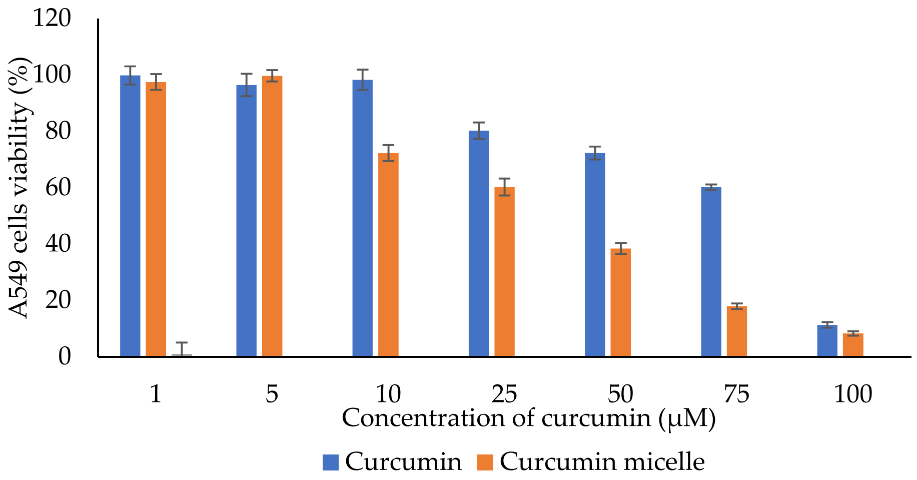

Figure S2: Cell viability of human non-small cell lung cancer cell line (A549) after 24 h treatment with various concentrations of curcumin and curcumin loaded QCS-Vani imine nano-micelles. All the values are expressed as mean ± standard deviation (n = 3) of curcumin concentrations.

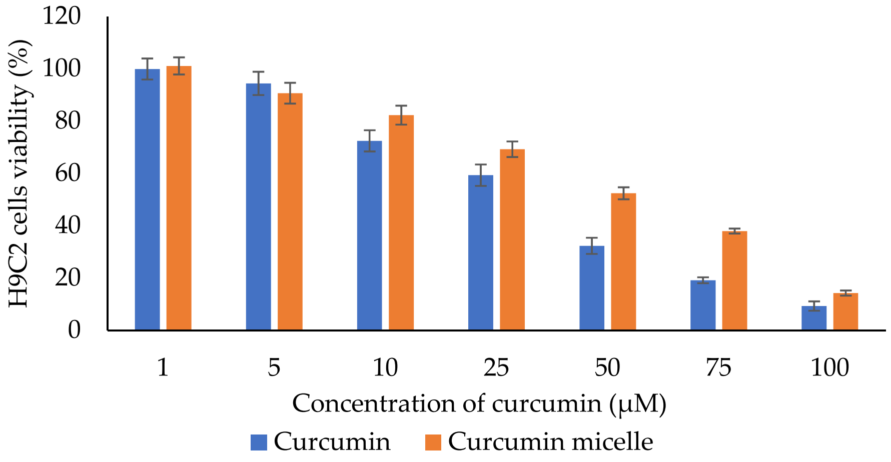

Figure S3: Cell viability of rat cardiomyocytes cell line (H9C2) after 24 h treatment with various concentrations of curcumin and curcumin loaded QCS-Vani imine nano-micelles. All the values are expressed as mean ± standard deviation (n = 3) of curcumin concentrations.

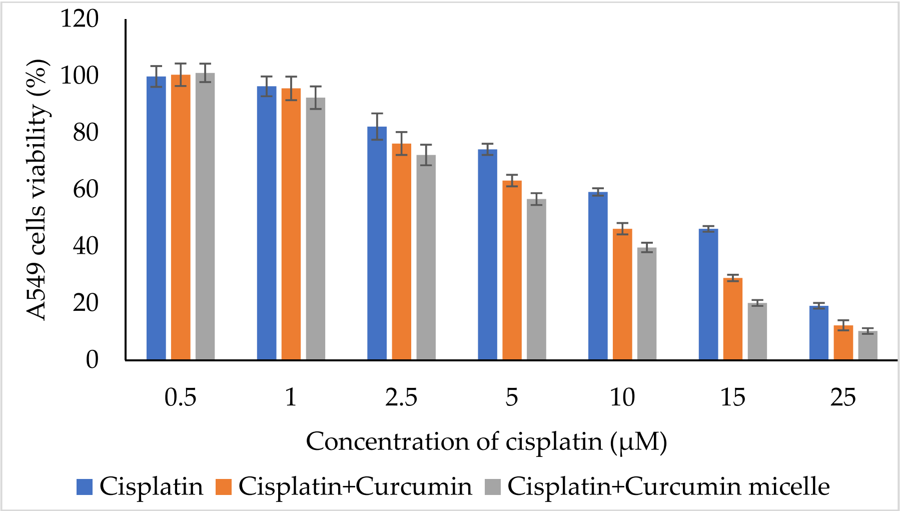

Figure S4: Cell viability of human non-small cell lung cancer cell line (A549) after 24 h treatment with various concentrations of cisplatin (in blue), cisplatin co-treatment with curcumin at 5 µM (IC_5) (in orange), cisplatin co-treatment with curcumin loaded QCS-Vani imine at 5 µM (IC_5) (in grey). All the values are expressed as mean ± standard deviation (n = 3) of cisplatin concentrations (µM).

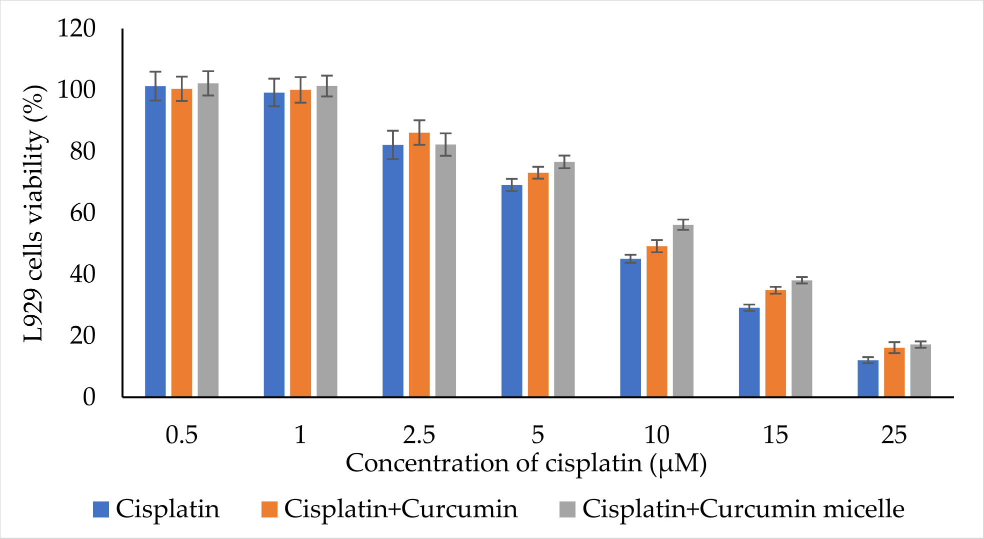

Figure S5: Cell viability of mouse fibroblast cell line (L929) after 24 h treatment with various concentrations of cisplatin (in blue), cisplatin co-treatment with curcumin at 5 μM (IC_5) (in orange), cisplatin co-treatment with curcumin loaded QCS-Vani imine at 5 μM (IC_5) (in grey). All the values are expressed as mean ± standard deviation (n = 3) of cisplatin concentrations (μM).

Tables

Table S1. Concentration ratios of cisplatin to curcumin when various concentrations of cisplatin co-treatment with curcumin at 5 μM (IC_5) and with curcumin loaded QCS-Vani imine at 5 μM (IC_5).

**Concentration of cisplatin (μM)**	**Concentration of pure curcumin (μM)**	**Conc. of curcumin in micelles (μM)**	**Concentration ratios**	
			**Cisplatin to pure curcumin**	**Cisplatin to curcumin in micelles**
0.5	5	5	0.1	0.1
1	5	5	0.2	0.2
2.5	5	5	0.5	0.5
5	5	5	1	1
10	5	5	2	2
15	5	5	3	3
25	5	5	5	5
